# Road avoidance and its energetic consequences for reptiles

**DOI:** 10.1002/ece3.5515

**Published:** 2019-08-13

**Authors:** James E. Paterson, James Baxter‐Gilbert, Frederic Beaudry, Sue Carstairs, Patricia Chow‐Fraser, Christopher B. Edge, Andrew M. Lentini, Jacqueline D. Litzgus, Chantel E. Markle, Kassie McKeown, Jennifer A. Moore, Jeanine M. Refsnider, Julia L. Riley, Jeremy D. Rouse, David C. Seburn, J. Ryan Zimmerling, Christina M. Davy

**Affiliations:** ^1^ Environmental and Life Sciences Program Trent University Peterborough ON Canada; ^2^ Department of Botany and Zoology Centre for Invasion Biology Stellenbosch University Stellenbosch Western Cape South Africa; ^3^ Environmental Studies and Geology Division Alfred University Alfred NY USA; ^4^ Ontario Turtle Conservation Centre Selwyn ON Canada; ^5^ Department of Biology McMaster University Hamilton ON Canada; ^6^ Canadian Forest Service Natural Resources Canada Fredericton NB Canada; ^7^ Toronto Zoo Toronto ON Canada; ^8^ Department of Biology Laurentian University Sudbury ON Canada; ^9^ School of Geography and Earth Sciences McMaster University Hamilton ON Canada; ^10^ Biology Department Grand Valley State University Allendale MI USA; ^11^ Department of Environmental Sciences University of Toledo Toledo OH USA; ^12^ Department of Botany and Zoology Stellenbosch University Stellenbosch Western Cape South Africa; ^13^ Parry Sound District Office Ontario Ministry of Natural Resources and Forestry Parry Sound ON Canada; ^14^ Seburn Ecological Services Ottawa ON Canada; ^15^ Canadian Wildlife Service Environment and Climate Change Canada Gatineau QC Canada; ^16^ Wildlife Research and Monitoring Section Ontario Ministry of Natural Resources and Forestry Peterborough ON Canada

**Keywords:** Blanding's turtle, eastern massasauga, energetics, movement ecology, road ecology

## Abstract

Roads are one of the most widespread human‐caused habitat modifications that can increase wildlife mortality rates and alter behavior. Roads can act as barriers with variable permeability to movement and can increase distances wildlife travel to access habitats. Movement is energetically costly, and avoidance of roads could therefore impact an animal's energy budget. We tested whether reptiles avoid roads or road crossings and explored whether the energetic consequences of road avoidance decreased individual fitness. Using telemetry data from Blanding's turtles (*Emydoidea blandingii*; 11,658 locations of 286 turtles from 15 sites) and eastern massasaugas (*Sistrurus catenatus*; 1,868 locations of 49 snakes from 3 sites), we compared frequency of observed road crossings and use of road‐adjacent habitat by reptiles to expected frequencies based on simulated correlated random walks. Turtles and snakes did not avoid habitats near roads, but both species avoided road crossings. Compared with simulations, turtles made fewer crossings of paved roads with low speed limits and more crossings of paved roads with high speed limits. Snakes made fewer crossings of all road types than expected based on simulated paths. Turtles traveled longer daily distances when their home range contained roads, but the predicted energetic cost was negligible: substantially less than the cost of producing one egg. Snakes with roads in their home range did not travel further per day than snakes without roads in their home range. We found that turtles and snakes avoided crossing roads, but road avoidance is unlikely to impact fitness through energetic expenditures. Therefore, mortality from vehicle strikes remains the most significant impact of roads on reptile populations.

## 1 INTRODUCTION

Roads are one of the most widespread human‐caused modifications of habitats, and the global road network exceeds 21 million km of road (Meijer, Huijbregts, Schotten, & Schipper, 2018). The global road network is predicted to grow by more than 25 million km by 2050 (Laurance et al., 2014), and mitigating the environmental consequences of roads requires understanding how wildlife interact with roads. Roads have major consequences for wildlife (Forman & Alexander, 1998; van der Ree, Jaeger, van der Grift, & Clevenger, 2011), including vehicle strikes that cause mortality, and can lead to population declines (Fahrig & Rytwinski, 2009; Gibbs & Shriver, 2002; Row, Blouin‐Demers, & Weatherhead, 2007). For populations that persist in proximity to roads, roads may act as a movement barrier that alters behavior (Beyer et al., 2016; Schwab & Zandbergen, 2011; Shepard, Kuhns, Dreslik, & Phillips, 2008). Reduced movement of individuals across roads can fragment populations (Fahrig, 2003), reduce gene flow (Clark, Brown, Stechert, & Zamudio, 2010; Row, Blouin‐Demers, & Lougheed, 2010), alter habitat use in response to roads (Lamb et al., 2018), and increase individual energetic costs if accessing resources requires traveling longer distances (Lusseau, 2004). The complete impact of roads on wildlife needs to be understood to inform effective management and mitigation, because road avoidance might mitigate direct mortality of wildlife on roads, but could have less obvious, indirect effects on individual fitness. The proximate causes of road avoidance vary widely among systems. Road avoidance may be affected by, or independent of, noise from traffic (Bouchard, Ford, Eigenbrod, & Fahrig, 2009; Ford & Fahrig, 2008; McClure, Ware, Carlisle, Kaltenecker, & Barber, 2013), visual disturbance from vehicles (Forman & Alexander, 1998), differences in habitat quality (Ortega & Capen, 1999), or changes in temperature and moisture caused by roads (LeGros, Steinberg, & Lesbarrères, 2017). In addition, some species respond differently to road types based on surface (e.g., paved or gravel) and traffic volume (Brehme, Tracey, McClenaghan, & Fisher, 2013; Robson & Blouin‐Demers, 2013; Whittington, St. Clair, & Mercer, 2005). Some mechanisms causing road avoidance (e.g., noise) may extend beyond the roadside, causing wildlife to avoid habitats from a few meters to several kilometers from the road itself (Benítez‐López, Alkemade, & Verweij, 2010). Understanding the consequences of road avoidance behavior on fitness requires understanding the spatial extent of the effect.

Road avoidance can affect fitness by changing energetic expenditures of wildlife living close to roads. Some species reduce their movement as the footprint of human activity increases (Tucker et al., 2018). Conversely, habitat loss may force individuals to travel longer distances to acquire resources, especially when these increased movements are paired with road avoidance. The energetic cost of movement can be used to extrapolate the magnitude of this impact (Lusseau, 2004). Reproduction is energetically costly (Congdon & Tinkle, 1982; McNab, 2006; Olsson, Madsen, & Shine, 1997; Shine, 1980), and reproductive rates are often energy‐limited. Therefore, increased expenditure of energy on movement may reduce reproductive output. No study has measured the potential energetic consequences of road avoidance (that we are aware of) or examined road avoidance in reptiles at a landscape scale.

Road avoidance behavior has been documented in a range of taxa (Andrews & Gibbons, 2005; Dyer, O'Neill, Wasel, & Boutin, 2002; Laurance, Stouffer, & Laurance, 2004; Proulx, Fortin, & Blouin‐Demers, 2014; Robson & Blouin‐Demers, 2013; Shepard et al., 2013), but these previous studies focussed on single populations and did not estimate fitness costs from increases in energy expenditure. Studies based on a single site with only one road network and habitat configuration should be interpreted with caution, as they may not apply at broader geographical scales or different road densities. Studying road avoidance at broad spatial scales reduces the risk of road configuration being conflated with other habitat features. Further, linking road avoidance to energetic costs of movement bridges the gap between habitat use and fitness to estimate the consequences of roads.

Roads are especially detrimental to reptiles, many of which have slow life histories (Andrews & Gibbons, 2005; Gibbs & Shriver, 2002). Reptile populations are declining globally (Böhm et al., 2013), due in part to the demographic effects of road‐related habitat modification, fragmentation, and mortality from vehicle strikes (Row et al., 2007; Steen et al., 2006). Several studies have demonstrated that reptiles avoid crossing roads (Proulx et al., 2014; Robson & Blouin‐Demers, 2013; Siers, Savidge, & Reed, 2014), but these focus on particular sites and may not be generalizable to the broader landscape.

**Figure 1:**
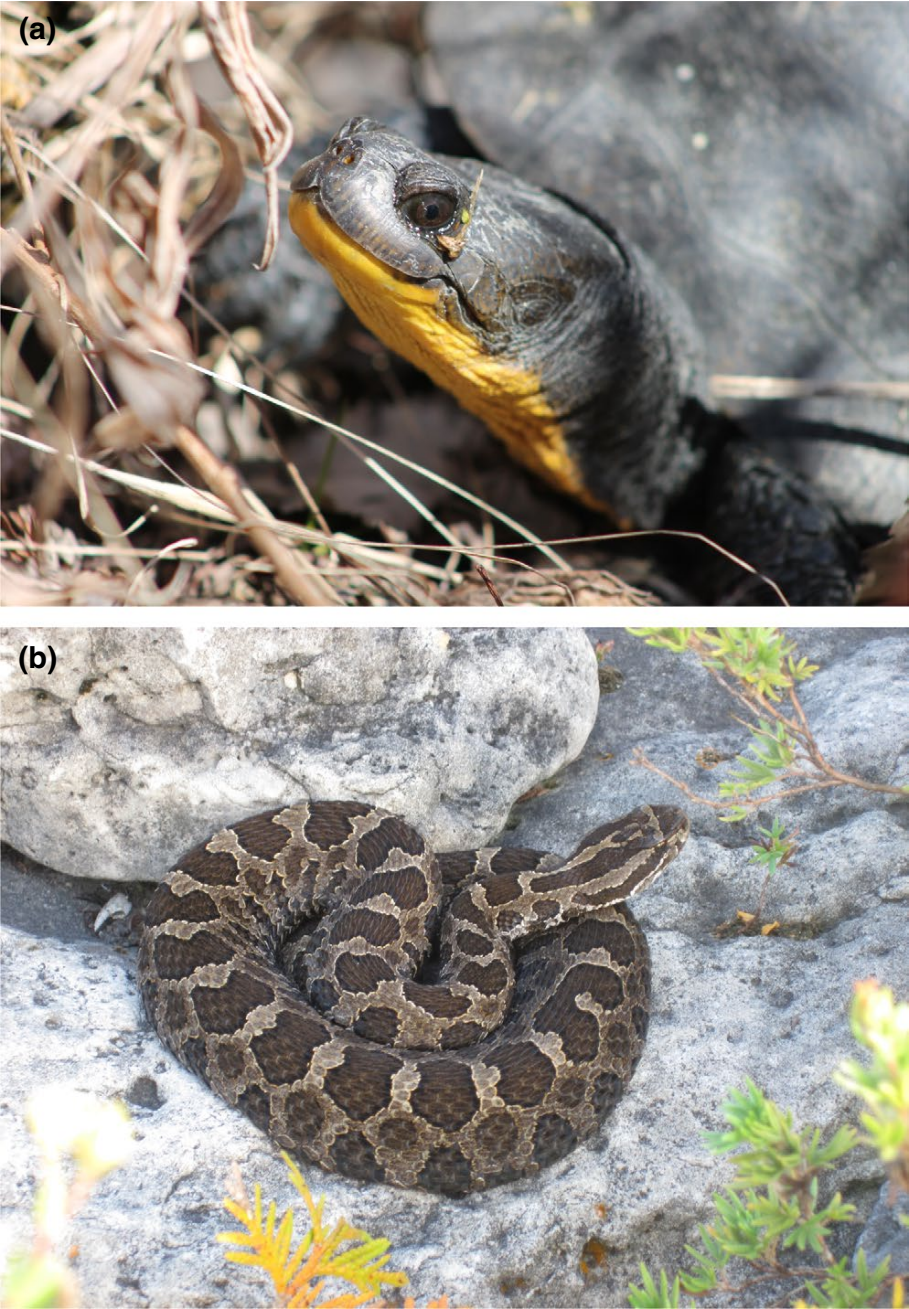
We studied road avoidance by (a) Blanding's turtle (*Emydoidea blandingii*) and (b) eastern massasauga (*Sistrurus catenatus*)

We tested whether reptiles avoid roads at broad spatial scales and whether road avoidance has significant energetic consequences using Blanding's turtle (*Emydoidea blandingii*) and eastern massasauga (*Sistrurus catenatus*) as model species (Figure 1). Blanding's turtle is listed as Endangered by the International Union for the Conservation of Nature (IUCN; van Dijk & Rhodin, 2011). Eastern massasauga is listed as Least Concern by the IUCN (Frost, Hammerson, & Santos‐Barrera, 2007), but is listed as Threatened (Great Lakes/St. Lawrence population) and Endangered (Carolinian population) in Canada by the Species at Risk Act (COSEWIC, 2013), and listed as Threatened under the United States Endangered Species' Act (Federal Register80, 2015). Road mortality is a major threat to both species (van Dijk & Rhodin, 2011; Frost et al., 2007). We aggregated telemetry data for Blanding's turtles and eastern massasaugas in North America and tested two indicators of potential road avoidance: frequency of road crossing and habitat selection relative to road proximity. We predicted that reptiles would cross roads less often than expected compared with simulated movement paths and that reptiles would spend more time in habitat further from roads than expected based on simulated locations. We also tested whether individuals with roads in their home range expended more energy on movement than individuals without roads in their home range.

## 2 MATERIALS AND METHODS

### 2.1 Tracking data

We aggregated telemetry data for Blanding's turtles and eastern massasaugas and retained individuals with ≥8 locations (Appendices S1–S4). The final dataset included 11,658 locations of 286 Blanding's turtles from 15 sites in Ontario, Minnesota, and Maine (Figure S1) collected between 2004 and 2017, and 1,868 locations of 49 eastern massasaugas from three sites in Ontario and Michigan (Figure S2) collected between 2001 and 2013. All analyses were done in R (R Core Team, 2018).

### 2.2 Road avoidance

We used public data on road networks in each jurisdiction (Geolibrary Maine, 2018; Michigan Geographic Framework, 2017; Minnesota Department of Transportation, 2012; Ontario Ministry of Natural Resources & Forestry, 2009), and local data collectors confirmed the accuracy of road networks at each site. Sites had a mean road density of 1.34 ± 0.29 km of road per km^2^ (Table S1). We counted the number of times the path of each tracked reptile crossed a road to quantify the minimum number of road crossings per individual.

We used simulated animal paths as null models to measure road avoidance. We simulated animal paths using the *adehabitatLT* package (Calenge, 2006) with constrained correlated random walk models, and we incorporated biologically relevant constraints to habitat selection (Proulx, Proulx, & Blouin‐Demers, 2013). Correlated random walk models simulate pathways based on the distribution of turning angles and step distances for the focal animal (Kareiva & Shigesada, 1983), so we added a habitat constraint to this base model. We defined habitat type using the Ontario Land Cover Compilation v.2.0 (Ontario Ministry of Natural Resources & Forestry, 2016; 15 m resolution) for Ontario sites and the National Land Cover Classification database (Homer et al., 2015; 30 m resolution) for sites in the United States. Blanding's turtles are semi‐aquatic (Beaudry, deMaynadier, & Hunter, 2008; Edge, Steinberg, Brooks, & Litzgus, 2010), so we constrained simulated paths to have the same proportion of locations in aquatic habitats as the observed locations (±1 *SD* of between‐individual variation = 29%). For example, simulated paths for a turtle with 70% of its real locations in aquatic habitats included 41%–99% locations in aquatic habitats. We included open water, shoreline, marsh, swamp, fen, and bog as aquatic habitat types in the Ontario land cover data. We included open water, woody wetlands, and emergent herbaceous wetlands as aquatic habitat in the United States land cover data. Eastern massasaugas use forested and prairie habitat in addition to wetlands (Moore & Gillingham, 2006) so we included additional habitat types in the habitat constraint (forest, alvar, and savannah in Ontario; barren rock, forest, and grassland in the United States; *SD* = 24%). For sites with exclusion fencing along roads (T13 and T14), we limited simulated paths to only cross roads at culverts or crossing structures and considered the fences to be complete barriers to movement. Fence boundaries and crossing structure locations were marked with a handheld GPS unit. We simulated 50 correlated random walk paths per individual using the same distribution of step distances and turning angles as the individual's actual path. Each simulated path started at the first location of the turtle or snake, and we counted the number of times each simulated path crossed a road to estimate expected number of crossings.

To compare the observed to the expected number of road crossings, we used generalized linear mixed‐effects models. We fit models with the *glmmTMB* package (Brooks et al., 2017) using number of road crossings as the response variable, the category (observed or expected) and number of days tracked (log‐transformed) as fixed effects, and individual nested within site as random effects. The response was zero‐inflated and right‐skewed, so we used a zero‐inflated negative binomial hurdle model. We constructed separate models for each species. If reptiles avoided crossing roads, then the paths of tracked reptiles would cross roads less often than simulated paths.

We also tested whether road crossing frequency depended on road type for data collected in Ontario by segregating observed road crossings by road class. The Maine, Michigan, and Minnesota road data did not include information on the road surface or speed limit. We classified Ontario roads as unpaved, paved with slow speeds (speed limit ≤ 60 km/hr), and paved with high speeds (speed limit > 60 km/hr). Paved roads with high speeds included major highways (>4 lanes, 100 km/hr) at some sites. We constructed separate generalized linear mixed‐effects models for each species (response variable: number of road crossings, fixed effects: log‐transformed number of days tracked, category as observed or expected, road class as unpaved, paved slow speed, or paved high speed, and the interaction between category and road class; random effects: individual nested within site). The number of crossings for simulated paths for each road class is correlated with the relative availability of each road class, so a significant interaction between road class and category would indicate that reptiles respond differently to different road classes compared with simulations. We fit the model with a zero‐inflated negative binomial hurdle structure.

To test whether reptiles avoided spending time near roads, we compared the minimum distance to roads for observed and expected animal locations. For each turtle, snake, or simulated reptile, we calculated the mean distance to the nearest road. We used generalized linear mixed‐effects models (response variable: mean distance to the nearest road; fixed effect: category as observed or expected; random effects: individual nested within site) to test whether reptiles were further from roads than expected. We used a negative binomial error distribution and constructed separate models for each species.

### 2.3 Energetic consequences

To test whether turtles and snakes with roads in their home ranges expend more or less energy on movement, we compared the movement costs of individuals with roads in their home range to individuals without roads in their home range. For each individual, we considered consecutive locations separated by >1 day and <14 days, and calculated the mean daily distance traveled (distance between locations/difference in time between locations). Using locations separated by more than 14 days could bias the mean daily distance downward (i.e., estimate shorter distances than were actually traveled) because of infrequent sampling. To confirm that our 1‐ to 14‐day window was not too wide, we also analyzed the data including only tracking intervals between 1 and 4 days. This resulted in similar qualitative and quantitative conclusions, so we included all tracking locations separated by 1–14 days in our analyses to increase the sample size and therefore the power of our analyses. We constructed home ranges with 100% minimum convex polygons (MCPs) and grouped individuals based on the presence or absence of any road segments within the home range. We used MCPs over kernel methods to keep home ranges continuous, and we used all points to avoid inadvertently biasing the results by excluding locations near roads. We constructed separate linear mixed‐effect models for turtles and snakes (response variable: mean daily distance traveled; fixed effect: roads in home range or roads absent from home range; random effect: site) to test whether reptiles with roads in their home range moved further per day.

To estimate the energy expenditure of a walking turtle, we used Zani and Kram's (2008) estimated costs of locomotion for ornate box turtles (*Terrapene ornata*) walking on a flat treadmill (no comparable studies exist for Blanding's turtles). These estimates represent a simplified energetic cost because free‐ranging individuals walk and swim in more complex habitats and are conservative (i.e., may under‐estimate actual energetic costs of potential road avoidance). We adjusted the mean cost of locomotion (8.0 J/kg*m) for ornate box turtles (Zani & Kram, 2008) to the mean mass of Blanding's turtles in our sample (1.35 kg; 10.8 J/m). We converted the estimated difference in daily movement between turtles with and without roads within their home ranges from the mixed‐effects model into Joules. To explore whether the estimated difference in energy spent moving might have fitness consequences, we extrapolated the predicted energetic difference per day over one active season (May 1 to September 1) and divided this prediction by the estimated energetic investment per egg (Congdon & van Loben Sels, 1991; MacCulloch & Weller, 1988; Rowe, 1992).

To estimate the energetic costs of potential road avoidance on snakes, we used the cost of locomotion for racers (*Coluber constrictor*; Walton, Jayne, & Bennett, 1990). The only study of movement costs on rattlesnakes was for sidewinders (*Crotalus cerastes*; Secor, Jayne, & Bennett, 1992). Sidewinding is more efficient than slithering; to be conservative, we used the higher estimate for racers. We adjusted the cost of slithering in racers (23.11 J/kg*m) to the mean mass of eastern massasaugas in our sample (223 g; 5.15 J/m) and converted the estimated difference in daily movement between snakes with and without roads within their home range to Joules. To explore the potential fitness consequences of increased movement, we extrapolated the predicted energetic difference of each day over one active season (May 1 to September 1) and divided this prediction by the estimated energetic investment per neonate snake (Jellen & Kowalski, 2007; Dyke & Beaupre, 2011).

## 3 RESULTS

### 3.1 Road avoidance

**Figure 2:**
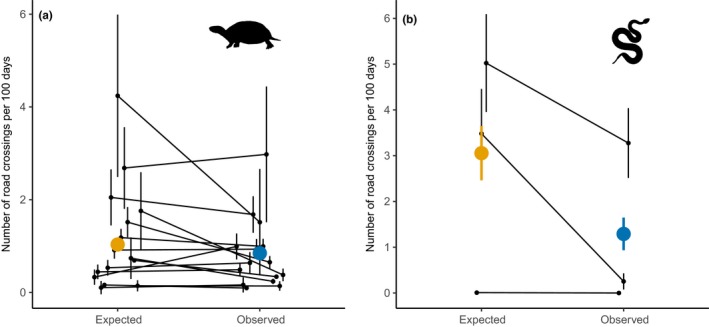
The observed number of road crossings/100 days (±*SE*) was (a) lower than expected for Blanding's turtle (*Emydoidea blandingii,* *n* = 286) paths at 15 sites and (b) lower for observed than expected for eastern massasauga (*Sistrurus catenatus,* *n* = 49) paths at three sites. Black lines connect the mean number of crossings from expected (based on 50 simulations per animal) to observed at each site. Colored points and lines represent the mean and *SE*

Road crossing frequency in Blanding's turtles varied widely among individuals (mean crossings/100 days = 0.85, median = 0.25, range = 0–17.65). Turtles crossed roads less frequently than predicted by simulated paths (mean crossings/100 days = 1.04, median = 0.41, range = 0–48.53; χ*^2^* = 16.44, *p* < .001, Figure 2a). The hurdle model, including random intercepts for each individual and site, predicted that turtles undertook 0–2 fewer road crossings per 100 days than simulated paths.

For the Ontario data, the number of real and simulated Blanding's turtle road crossings differed among road classes (main effect of road class; χ*^2^* = 2,916.6, *p* < .001; Figure S3). There was an interaction between road class and category of observed versus expected (χ*^2^* = 32.1, *p* < .001). Turtles crossed paved roads with low speed limits (0.06 ± 0.002 crossings/100 days) less than expected (0.12 ± 0.004 crossings/100 days; Wald *z*‐score = 2.35, *p* = .02; Figure S3). There were 21 turtles (10%) that crossed paved roads with low speed limits at least once. Turtles crossed paved roads with high speed limits (0.23 ± 0.01 crossings/100 days) more than expected (0.20 ± 0.01 crossings/100 days; Wald *z*‐score = 4.31, *p* < .001). There were 28 turtles (13%) that crossed paved roads with high speed limits at least once. There was no difference in crossing frequency between observed (0.39 ± 0.01 crossings/100 days) and expected (0.43 ± 0.02 crossings/100 days) turtle paths on unpaved roads (Wald *z*‐score = 0.35, *p* = .73). There were 69 turtles (33%) that crossed unpaved roads at least once.

Number of road crossings varied among individual eastern massasaugas (mean crossings/100 days = 1.3, median = 0; range = 0–12.0). Snakes made fewer road crossings than predicted by simulated paths (mean crossings/100 days = 3.1, median = 1.4, range = 0–29.6; χ*^2^* = 39.44, *p* < .001, Figure 2b). The model, including random intercepts for each individual and site, predicted that snakes at the three sites made 0–6 fewer crossings per 100 days than expected based on simulated paths.

In the Ontario dataset where roads were grouped by surface and speed limit, the number of road crossings for observed and simulated snake paths differed among road classes (effect of road class; χ*^2^* = 1,687.4, *p* < .0001). Snakes made fewer road crossings than expected (χ*^2^* = 24.2, *p* < .0001), and snakes crossed all road types less often than expected (Figure S3). There was no significant interaction between road class and category of observed versus expected on crossing frequency (χ*^2^* = 2.30, *p* = .68), and the difference in road crossing frequency between observed and expected values was not affected by the type of road.

**Figure 3:**
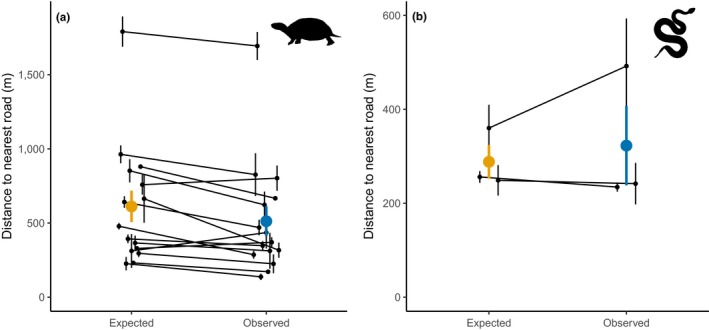
Mean distance (±*SE*) to the nearest road for (a) Blanding's turtles (*Emydoidea blandingii*, *n* = 286) at 15 sites and (b) eastern massasauga (*Sistrurus catenatus*, *n* = 49) at three sites. Expected locations were based on 50 simulations/individual, and observed locations were based on tracking of free‐ranging individuals. Observed turtle locations were closer to roads than expected, while snake locations were not different than expected values. Black lines connect the mean expected and observed crossings at each site. Colored points and lines represent the overall mean and SE for the entire dataset

Blanding's turtle locations relative to roads varied widely (range in mean distance to road: 28–2,691 m). Turtles were closer to the nearest road (514 ± 29 m) than expected (612 ± 28 m) based on simulations (χ*^2^* = 56.93, *p* < .0001; Figure 3a). Eastern massasauga locations relative to road also varied widely (range in mean distance to roads = 46–1,548 m). There was no difference in the distance from observed and simulated snake locations to the nearest road (observed: 327 ± 42 m; expected: 289 ± 22 m; χ*^2^* = 3.10, *p* = .08; Figure 3b).

### 3.2 Energetic consequences

**Figure 4:**
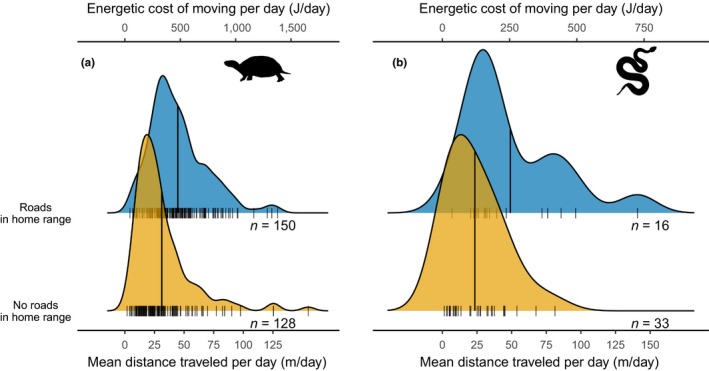
(a) The mean (vertical lines) distance traveled and energetic cost of moving per day for Blanding's turtles (*Emydoidea blandingii*, *n* = 278) was higher for turtles with roads in their home ranges. (b) The mean (vertical lines) distance traveled and energetic cost of moving per day for eastern massasaugas (*Sistrurus catenatus*, *n* = 49) did not differ between snakes with and without roads in their home ranges

Turtles with roads in their home range had a mean 0.85 ± 0.21 km of road within their home range. Snakes with roads in their home range had a mean 0.38 ± 0.08 km of road within their home range. Blanding's turtles with roads in their home ranges moved further per day (45 ± 2 m) than turtles without roads in their home range (31 ± 2 m, *F* = 16.86, *df* = 1, 269, *p* < .0001; Figure 4a), and turtle movements increased with cumulative road distance within their home range (*F* = 27.35, *df* = 1, 125, *p* < .0001, Figure S4). This translated to an increase of 136 J per day in energy expenditure or 16.8 kJ over an active season from May 1 to September 1. Producing one egg requires 88 kJ, assuming 7.05 kJ/g of egg (Congdon & Tinkle, 1982) and a mean egg mass of 12.5 g. Blanding's turtle typically lays 8–15 eggs/clutch (Congdon & van Loben Sels, 1991; MacCulloch & Weller, 1988; Rowe, 1992). Therefore, the predicted increase in annual energy expenditure represents 19% of the energy required to produce one egg.

Eastern massasaugas with roads in their home range did not move significantly further per day than snakes without roads in their home range (*F* = 1.54, *df* = 1, 37, *p* = .22; Figure 4b). Snakes moved similar distances per day at site S2 (roads in home range: 40 ± 6 m; no roads in home range: 34 ± 4 m) and site S3 (roads in home range: 50 ± 10 m; no roads in home range: 38 ± 15 m; Figure S5). Snakes at S1 moved much less per day (no roads in home range: 8 ± 1 m; there were no snakes with roads in their home range at this site). Daily movement of snakes did not increase with cumulative road distance within their home range (*F* = 1.93, *df* = 1, 13, *p* = .19, Figure S4), but we had low power to detect an effect. On average, snakes with roads in their home ranges used an estimated additional 73 J/day, or 9.0 kJ per active season. Producing one neonate snake requires 65 kJ, assuming 6.75 kJ/g of snake (Dyke & Beaupre, 2011) and a mean neonate mass of 9.6 g (Jellen & Kowalski, 2007). Therefore, the predicted increase in annual energy expenditure represents 14% of the energy required to produce one neonate or 1% of a mean litter of 13 neonates (Parent & Weatherhead, 2000).

## 4 DISCUSSION

This study is the first to evaluate road avoidance behavior of reptiles at large spatial scales and the first to estimate energetic consequences of road avoidance in reptiles. Our results suggest that Blanding's turtles and eastern massasaugas avoid crossing roads, but do not avoid habitats adjacent to roads. Turtles expend more energy on movement in home ranges containing more roads, but the increased energetic expenditure is small compared with energetic investment in reproduction. Overall, our results suggest that reptiles avoid road crossings but not roads per se and that the presence of roads impacts movement of reptiles. However, the additional energy expenditure of reptiles interacting with roads is unlikely to negatively affect fitness through reduced reproductive output, and road crossing avoidance may have positive effects by reducing mortality risk. Mitigation of road impacts on reptile populations should continue to focus on reducing mortality from vehicle strikes.

Avoidance of road crossings by Blanding's turtles and eastern massasaugas is consistent with previous work examining road avoidance behavior in reptiles (Andrews & Gibbons, 2005; Proulx et al., 2014; Robson & Blouin‐Demers, 2013; Shepard et al., 2008; Siers et al., 2014). Where roads act as strong barriers, they may also fragment populations and have genetic consequences, especially for small populations (Clark et al., 2010; Row et al., 2010), but our data do not suggest a complete avoidance of road crossing. We found that reptiles crossed roads less frequently than expected (i.e., avoid some road crossings), but the reduction in crossings was small (0–6 crossings per individual per 100 days). This small reduction in crossings is unlikely to have significant energetic costs, but may substantially increase lifetime fitness if the probability of surviving a road crossing is low (Aresco, 2005).

Turtles responded differently to road type, showing the greatest avoidance of road crossing on paved roads with low speed limits, and snakes equally avoided crossing all road types. The only other study (that we are aware of) to examine road avoidance in Blanding's turtles found that turtles at a single study site avoided crossing both paved and unpaved roads (Proulx et al., 2014), but did not distinguish between speed limits on paved roads. Previous studies found that lizards, small mammals, and snakes are more likely to cross unpaved roads (Brehme et al., 2013; Robson & Blouin‐Demers, 2013), and increased traffic decreases the probability of crossing a road (Brehme et al., 2013). Crossing rates over unpaved roads were higher than other road classes for turtles, but not for snakes. Avoiding open areas such as roads is likely an anti‐predator behavior, and some species of small mammals, amphibians, and snakes avoid roads regardless of traffic levels (Andrews & Gibbons, 2005; Bouchard et al., 2009; McGregor, Bender, & Fahrig, 2008). Turtles crossed paved roads with high speed limits more than expected, which was opposite of our prediction. It is possible there were unmeasured correlations between road type and adjacent habitat that affected our interpretation of crossing frequencies over different road types. Our results suggest that Blanding's turtles and eastern massasaugas avoid crossing roads, but turtles more strongly avoided crossing paved roads with low speed limits and turtles crossed paved roads with high speed limits more than expected.

Neither turtles nor snakes showed avoidance of roadside habitats, and Blanding's turtles were found closer to roads than expected. Roadside habitats may act as ecological traps, where otherwise suitable habitats are associated with increased mortality risk (Kristan, 2003) or where individual fitness is indirectly affected by proximity to roads. Turtles may be attracted to roadsides by canopy openings that create basking and nesting sites.

Blanding's turtles with roads in their home range experienced a predicted 41% increase in energy expenditure (per year) compared with turtles without roads in their home range. However, this increase may not impact individual fitness, as it represents only 19% of the energy required to produce a single egg. Eastern massasaugas with roads in their home ranges also moved farther per day (predicted + 14 m/day) than snakes without roads in their home ranges. This difference was similar to the difference predicted for turtles (+13 m/day), but was not statistically greater than zero, possibly because we had lower power to detect statistical difference in the aggregated snake data (*n* = 33 and 16 snakes with and without roads in their home ranges, respectively). Increased movement in snakes near roads represented 13% of the energy investment for one neonate and 1% of the energy investment in an average litter. Overall, these results do not support the hypothesis that road avoidance places significant energetic costs on Blanding's turtles or eastern massasaugas, and roads are unlikely to indirectly impact individual fitness through increased energetic costs.

Our results raise further questions about indirect effects of roads on turtle and snake populations. We observed an association between roads and altered movement behavior in reptiles when roads were well established in their home ranges. A before‐after‐control‐impact tracking study of reptiles as roads are constructed could test whether turtles and snakes move more while adjusting to new roads, and whether this adjustment period may involve significant increases in energy expenditure. Also, the proximate cause of road avoidance by turtles and snakes has not been identified. Future work should use an experimental framework to test whether road crossing avoidance in reptiles is learned or instinctive and whether it reflects a response to traffic noise, visual disturbance, or other habitat alterations (e.g., temperature or moisture variation). Eastern massasaugas in Ontario tracked during and after road construction did not avoid roads that were still closed to public traffic, suggesting noise may be the mechanism that causes avoidance (Rouse, Willson, Black, & Brooks, 2011). This information is critical to mitigating effects of roads on reptiles because not all roads may be avoided to the same degree.

Roads have broad ecological effects through increases in mortality of wildlife from vehicle strikes (Gibbs & Shriver, 2002), decreased gene flow (Clark et al., 2010), and altered behavior and movement of some species (Dyer et al., 2002; Robson & Blouin‐Demers, 2013; this study). We found evidence that turtles and snakes alter movement behavior in response to roads, but road avoidance is unlikely to negatively affect fitness through increased energetic costs of movement. Thus, road mortality remains the most significant impact of roads on reptile populations, with some roads causing hundreds of reptile mortalities per year (Baxter‐Gilbert, Riley, Lesbarrères, & Litzgus, 2015; Teixeira, Coelho, Esperandio, & Kindel, 2013).

## CONFLICT OF INTEREST

The authors have no competing interests to declare.

## AUTHOR CONTRIBUTIONS

J.E.P, C.M.D, and J.R.Z formulated the study; J.B.‐G, F.B., S.C., P.C.‐F., C.B.E., A.M.L., J.D.L., C.E.M., K.M., J.A.M., J.M.R., J.L.R., J.D.R, and D.C.S. collected data; J.E.P. analyzed data; J.E.P. and C.M.D. led the writing of the manuscript. All authors contributed critically to the drafts and gave final approval for publication.

## Data Availability

All data used in this study can be found on the Dryad Data Repository: https://doi.org/10.5061/dryad.5tc40vg.
