# Supplementary material for: Road avoidance and its energetic consequences for reptiles
Source: Ecol Evol. 2019 Aug 13;9(17):9794–803. doi: 10.1002/ece3.5515 (PMC6745830; doi:10.1002/ece3.5515)
Supplement: Supplementary file 1 [file ECE3-9-9794-s001.docx]

**Supporting Information for: Paterson et al. “Road avoidance and its energetic consequences for reptiles”**

**Appendix S1. Study sites and sample sizes for tracking data**

**
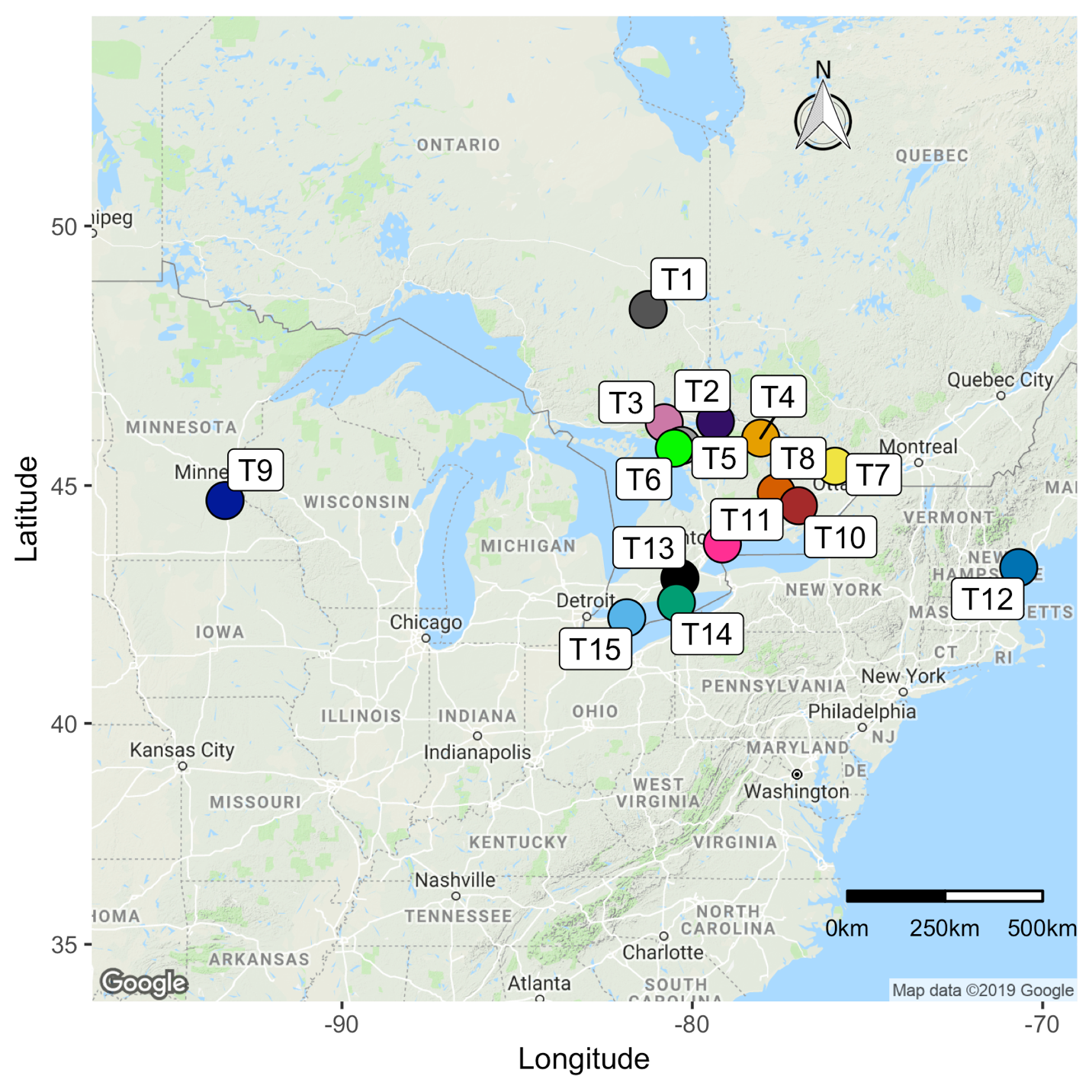
Figure S1.** Locations of 15 Blanding’s turtle (*Emydoidea blandingii*) study sites.

**
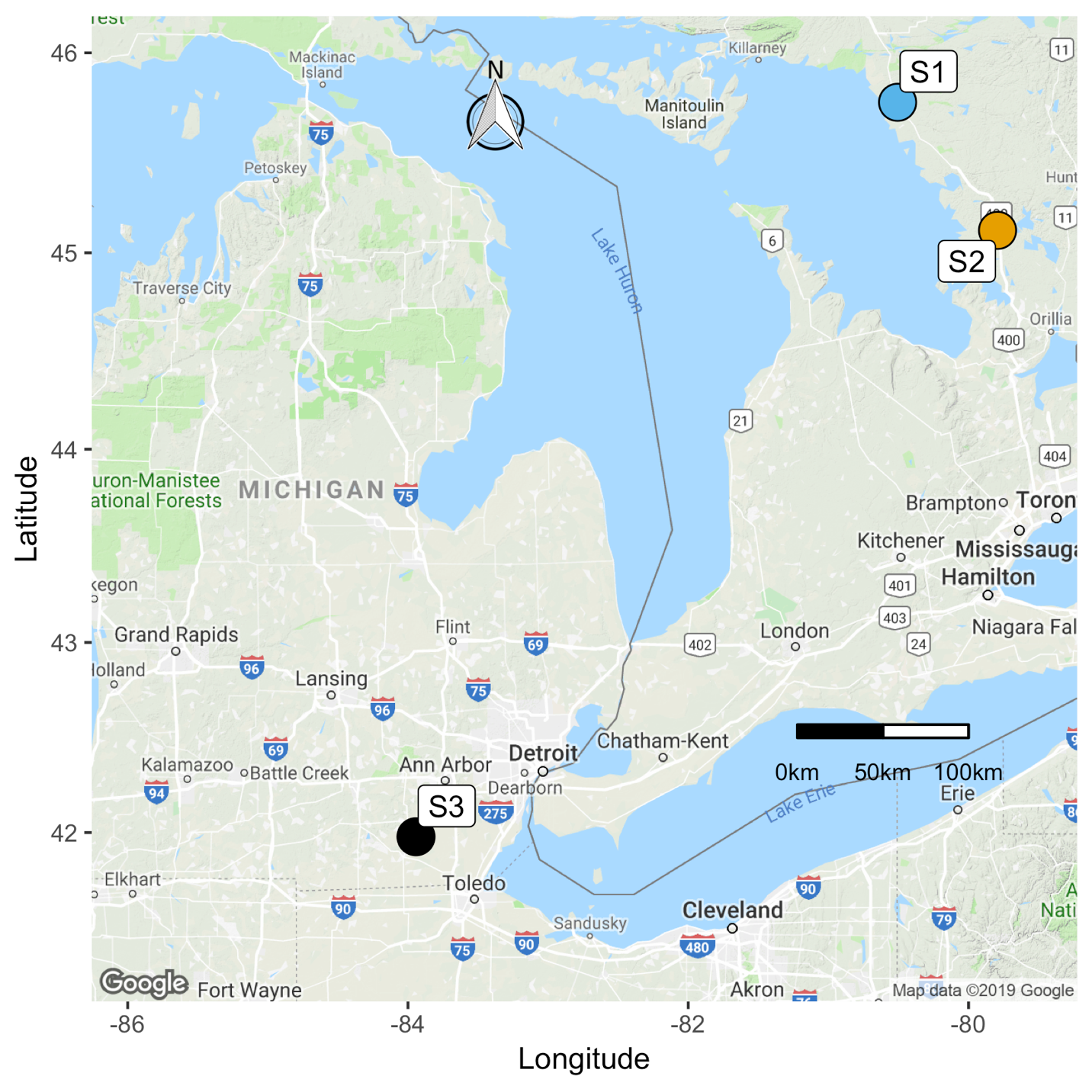
**

**Figure S2.** Locations of 3 eastern massasauga (*Sistrurus catenatus*) study sites.

**Table S1**. The number of animals and tracking locations of Blanding’s turtles (*Emydoidea blandingii*) at 15 study sites (T1 to T15) and eastern massasaugas (*Sistrurus catenatus*) at 3 study sites (S1 to S3) with varying levels of road density.

| Site | Number of animals | Number of locations | Road density  (km of road/km^2^) | Years | Mean days between relocations |
| --- | --- | --- | --- | --- | --- |
| T1 | 1 | 97 | 0.92 | 2010-2011 | 4.2 |
| T2 | 6 | 181 | 1.50 | 2006-2007 | 3.2 |
| T3 | 4 | 194 | 0.72 | 2011-2013 | 4.9 |
| T4 | 20 | 967 | 0.07 | 2014-2015 | 7.1 |
| T5 | 35 | 1958 | 0.80 | 2007 | 4.4 |
| T6 | 6 | 358 | 0.38 | 2004-2006 | 4.2 |
| T7 | 8 | 227 | 1.56 | 2012-2013 | 5.3 |
| T8 | 40 | 1590 | 0.65 | 2012 | 2.6 |
| T9 | 24 | 1071 | 3.73 | 2011-2013 | 1.8 |
| T10 | 21 | 494 | 0.33 | 2012 | 2.8 |
| T11 | 7 | 781 | 4.64 | 2003-2004 | 3.1 |
| T12 | 50 | 1943 | 1.98 | 2008-2011 | 5.6 |
| T13 | 19 | 813 | 1.71 | 2010-2012 | 4.1 |
| T14 | 28 | 686 | 0.67 | 2013-2017 | 7.6 |
| T15 | 17 | 298 | 0.35 | 2005-2015 | 3.3 |
| S1 | 18 | 482 | 0.34 | 2003-2004 | 4.5 |
| S2 | 17 | 1040 | 1.08 | 2004 | 2 |
| S3 | 14 | 346 | 2.64 | 2012-2013 | 2.5 |

**Appendix S2. Crossing frequency over different road classes for observed and simulated reptiles**

**
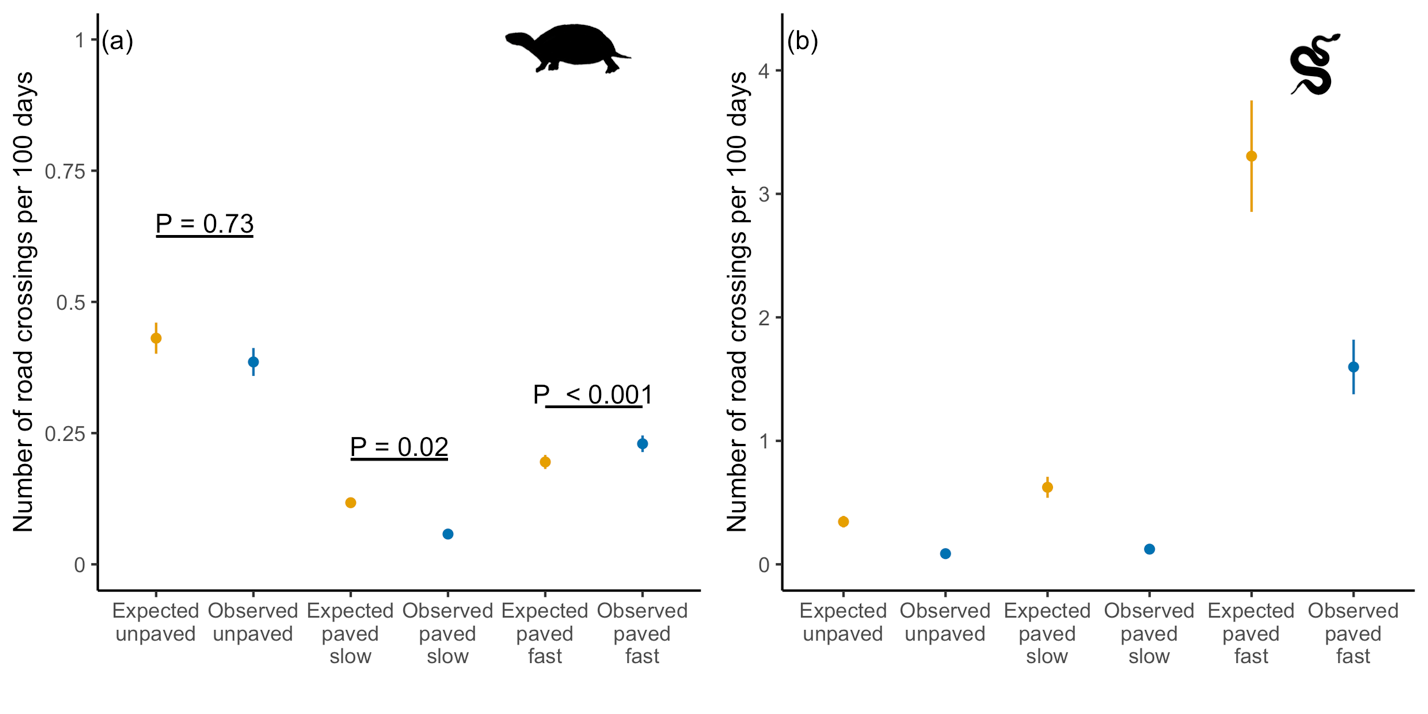
**

**Figure S3**. (a) The mean number of road crossings (± SE) for observed (blue) and expected (gold) Blanding’s turtle (*Emydoidea blandingii,* n = 212) paths at 13 sites for three road classes in Ontario. *P* values are for Wald z-scores from the mixed-effects zero-inflated hurdle model. (b) The mean number of road crossings (± SE) for all three road classes was lower for observed than expected in eastern massasauga (*Sistrurus catenatus,* n = 49) paths at two sites in Ontario.

**Appendix S3. Distance travelled per day and length of roads within home ranges
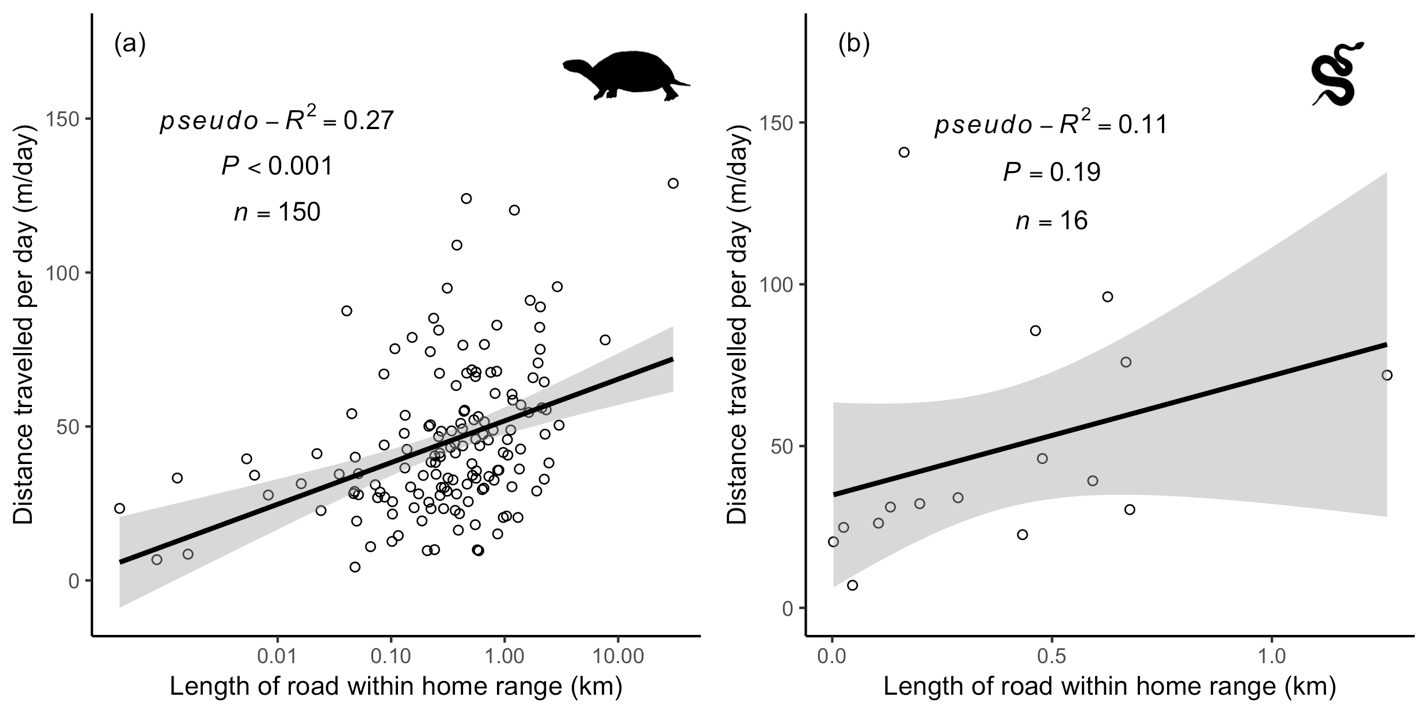
**

**Figure S4.** The relationship between the length of road within a home range (km) and the distance travelled per day (m/day) by (a) Blanding’s turtles (*Emydoidea blandingii*; n = 150) and (b) eastern massasaugas (*Sistrurus catenatus*; n = 16). Linear trends are shown in black (± SE in gray).

**Appendix S4. The mean distance travelled per day by eastern massasaugas**

**
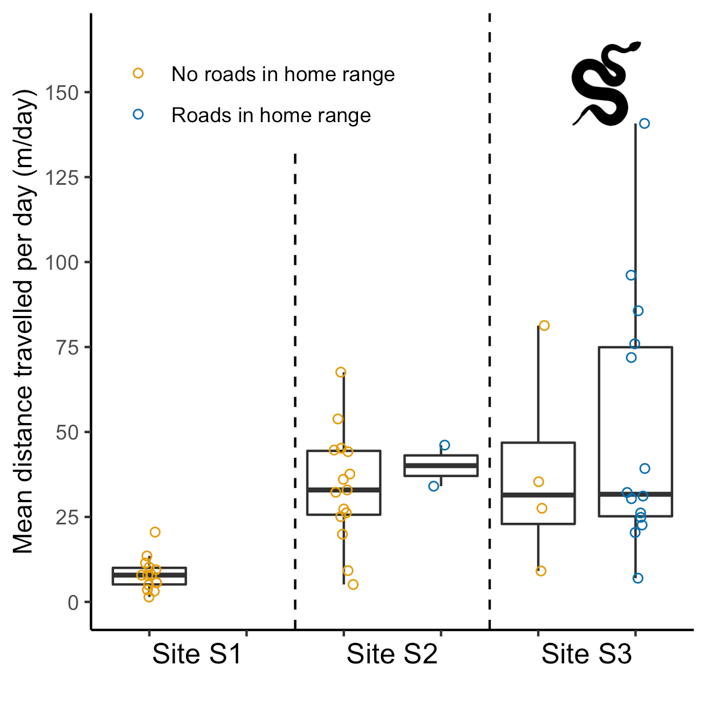
**

**Figure S5.** The mean distance travelled per day did not differ between eastern massasaugas (*Sistrurus catenatus*, n = 49) with and without roads in their home range at three study sites (S1, S2, and S3). Horizontal lines are medians and boxes represent interquartile limits.
